# Long-Term Activation of Group I Metabotropic Glutamate Receptors Increases Functional TRPV1-Expressing Neurons in Mouse Dorsal Root Ganglia

**DOI:** 10.3389/fncel.2016.00079

**Published:** 2016-03-31

**Authors:** Takayoshi Masuoka, Makiko Kudo, Junko Yoshida, Takaharu Ishibashi, Ikunobu Muramatsu, Nobuo Kato, Noriko Imaizumi, Matomo Nishio

**Affiliations:** ^1^Department of Pharmacology, School of Medicine, Kanazawa Medical UniversityUchinada, Japan; ^2^Department of Pharmacology, School of Nursing, Kanazawa Medical UniversityUchinada, Japan; ^3^Department of Physiology I, School of Medicine, Kanazawa Medical UniversityUchinada, Japan

**Keywords:** metabotropic glutamate receptors, TRPV1, TRPA1, dorsal root ganglion, heat hyperalgesia

## Abstract

Damaged tissues release glutamate and other chemical mediators for several hours. These chemical mediators contribute to modulation of pruritus and pain. Herein, we investigated the effects of long-term activation of excitatory glutamate receptors on functional expression of transient receptor potential vaniloid type 1 (TRPV1) in dorsal root ganglion (DRG) neurons and then on thermal pain behavior. In order to detect the TRPV1-mediated responses in cultured DRG neurons, we monitored intracellular calcium responses to capsaicin, a TRPV1 agonist, with Fura-2. Long-term (4 h) treatment with glutamate receptor agonists (glutamate, quisqualate or DHPG) increased the proportion of neurons responding to capsaicin through activation of metabotropic glutamate receptor mGluR1, and only partially through the activation of mGluR5; engagement of these receptors was evident in neurons responding to allylisothiocyanate (AITC), a transient receptor potential ankyrin type 1 (TRPA1) agonist. Increase in the proportion was suppressed by phospholipase C (PLC), protein kinase C, mitogen/extracellular signal-regulated kinase, p38 mitogen-activated protein kinase or transcription inhibitors. Whole-cell recording was performed to record TRPV1-mediated membrane current; TRPV1 current density significantly increased in the AITC-sensitive neurons after the quisqualate treatment. To elucidate the physiological significance of this phenomenon, a hot plate test was performed. Intraplantar injection of quisqualate or DHPG induced heat hyperalgesia that lasted for 4 h post injection. This chronic hyperalgesia was attenuated by treatment with either mGluR1 or mGluR5 antagonists. These results suggest that long-term activation of mGluR1/5 by peripherally released glutamate may increase the number of neurons expressing functional TRPV1 in DRG, which may be strongly associated with chronic hyperalgesia.

## Introduction

Inflammation and peripheral nerve injury cause release of glutamate and other neurotransmitters/chemical mediators from sensory neurons, glia, and damaged cells in the spinal cord and peripheral tissue in rodents (Omote et al., 1998; Lawand et al., 2000; Liu and Salter, 2010; Miller et al., 2011). These mediators elicit firing in nociceptive neurons resulting in the induction of inflammatory and neuropathic pain. A similar phenomenon has been reported in humans: glutamate concentration in the synovial fluid of synovitis patients was 54 times higher than in non-arthritic control subjects (McNearney et al., 2000).

Immunohistochemical studies have shown that sensory neurons express various glutamate receptors, such as N-methyl-D-aspartate (NMDA) and alpha-amino-3-hydroxy-5-methylisoxazole-4-propionic acid (AMPA) receptors in rat glabrous skin (Carlton et al., 1995). In addition, group I metabotropic glutamate receptors (mGluRs) consisting of two subtypes, mGluR1 and mGluR5, are also expressed in cell bodies of dorsal root ganglion (DRG) neurons and unmyelinated afferents in peripheral tissue (Bhave et al., 2001). Systemic injection of an mGluR1/5 agonist leads to an increase in sensitivity to noxious heat, or thermal hyperalgesia, while selective mGluR1 or mGluR5 antagonists attenuate inflammatory pain (Bhave et al., 2001; Zhou et al., 2001).

Recent studies indicate that mGluR5 in DRG neurons contributes to activating and/or sensitizing transient receptor potential vaniloid type 1 (TRPV1) channel. TRPV1 channel is activated by capsaicin, noxious heat (>43°C), low pH or various lipids, resulting in sensitization of sensory neurons in acute pain. TRPV1 activation via mGluR5 has been shown to produce an elevation in intracellular calcium concentration in a small number of rat and mouse DRG neurons (Crawford et al., 2000; Hu et al., 2002; Kim et al., 2009). TRPV1 activation is mediated by the production of prostaglandins via the phospholipase C (PLC) cascade in peripheral terminals of DRG neurons (Hu et al., 2002) and the production of diacylglycerol in central terminals (Kim et al., 2009). In addition, our previous work has revealed that mGluR5 biphasically modulates the intracellular calcium response induced by capsaicin, a TRPV1 agonist, in DRG neurons; mGluR5 activation potentiates the TRPV1-mediated intracellular calcium response, whereas subsequent cessation of mGluR5 activation depresses it, thus contributing to heat hyper- and hypoalgesia (Masuoka et al., 2015a,b).

Previous studies on the activation of mGluR5 receptors in the context of pain or inflammation have focused on short-term activation for a period of 5–20 min. However, glutamate remains elevated for much longer periods during inflammation. For example, a recent microdialysis study revealed that inflammation induced by formalin elevated extracellular glutamate concentration in the hind paw for over 3 h (Omote et al., 1998). In the present study, we show the long-term effects of group I mGluRs on DRG neurons. In particular, it was tested whether the proportion of functional TRPV1-expressing neurons are changed, thereby contributing to chronic heat hyperalgesia.

## Materials and Methods

All animal procedures were approved by the Ethics Committee of Kanazawa Medical University and animals were treated humanely, in accordance with the “Guiding Principles for the Care and Use of Laboratory Animals” set by the Japanese Pharmacological Society.

### Preparation of Primary Cultures

Culture preparation was conducted as described previously (Masuoka et al., 2015b). Six- to fourteen-day-old C57BL/6J mice purchased from SLC (Shizuoka, Japan) were anesthetized with inhalation of isoflurane (Escain^®^, Mylan Inc., Cecil Township, PA, USA). DRG were rapidly dissected in ice-cold Ca^2+^/Mg^2+^-free artificial cerebrospinal fluid (ACSF: 138.6 mM NaCl, 3.35 mM KCl, 21 mM NaHCO_3_, 9.9 mM glucose, 0.6 mM NaH_2_PO_4_, 2.5 mM CaCl_2_, and 1 mM MgCl_2_) gassed with a mixture of 95% O_2_ and 5% CO_2_ (pH 7.4). DRG neurons were dissociated following treatment with 0.1% type II collagenase (240–265 U/mg; Worthington Biochemical Co., Lakewood, NJ, USA), 0.1% trypsin (Gibco, San Diego, CA, USA), and 0.01% DNase I (Sigma, St. Louis, MO, USA) in Ca^2+^/Mg^2+^-free ACSF and shaken (35 cycle/min) in a water bath at 37°C for 30 min. Cells were gently triturated in Dulbecco’s Modified Eagle Medium (DMEM; Sigma) containing 10% horse serum (Gibco), 5% fetal calf serum (Gibco), and 1% penicillin-streptomycin (Wako, Osaka, Japan). Dispersed cells were passed through a 100-μm Cell Strainer (BD Biosciences, San Jose, CA, USA) and the filtered cells were seeded on glass coverslips (13-mm diameter) coated with poly-L-lysine (Matsunami Glass Ind., Osaka, Japan). Calcium imaging and whole-cell patch clamp recording were performed 24–48 h after dissociation.

### Calcium Imaging

Changes in intracellular calcium were measured with a fluorescent calcium indicator, as described previously (Yoshida et al., 2012; Masuoka et al., 2015b). For microscopic fluorometric measurement, cultured DRG neuronal cells were washed twice with ACSF and incubated for 45 min in the CO_2_ incubator (37 ± 2°C) in a solution of 3 μM of Fura-2-acetoxymethyl ester (Fura-2 AM; Dojindo Laboratories, Kumamoto, Japan) and 0.005% Cremophor EL (Sigma). After incubation, cells were washed in ACSF for 30 min and culture dishes were placed on the stage of an inverted microscope (ECLIPSE TE 300, Nikon, Tokyo, Japan) equipped with a 20× S-fluor objective. Fluorescence images were recorded and analyzed using a video image analysis system (ARGUS/HiSCA, Hamamatsu Photonics, Hamamatsu, Japan). Experimental agents were dissolved in ACSF and delivered by incubation in the CO_2_ incubator and/or continuous perfusion in the recording chamber (2 mL/min) with a peristaltic pump. Image pairs were captured at 10 s intervals. Fura-2 fluorescence was recorded at an emission wavelength of 510 nm by exciting Fura-2 at 340 and 380 nm. The 340 to 380 nm fluorescence ratio (F340/F380) was used as a parameter of intracellular calcium concentration. To prevent the variance of neuronal population in DRG culture, cultures made from same mice at the same time were used in control and comparison groups in one experiment. Experiments were performed 4–6 times for each drug condition, with simultaneous recording of 20–40 neurons per iteration.

### Neurons Viability Assay

To elucidate the neuronal death by glutamatergic drugs, cultured neurons were stained with a physiological solution containing 0.25% tripan blue (WAKO) for 5 min. Blue-stained and unstained neurons, which were recognized as dead and live neurons respectively, were counted in random fields under 200× magnification (DIAPHOT300, Nikon, Tokyo, Japan).

### Whole-Cell Patch Clamp Recording

Cultured neurons were plated onto coverslips, transferred to the recording chamber, and superfused with ACSF. Neurons were visually identified using a 60× microscope objective (DIAPHOT300, Nikon, Tokyo, Japan). Pipettes for whole-cell recordings were made from borosilicate glass capillaries (1.5 mM outer diameter; World Precision Instruments Inc., Sarasota, FL, USA). Patch pipettes (4–6 MΩ) were filled with an internal solution containing 120 mM KCH_3_SO_3_, 5 mM KCl, 10 mM K-EGTA, 5 mM Na-HEPES, 3 mM Mg-ATP, and 0.4 mM Na-GTP (pH 7.4). Series resistance was 8–20 MΩ, which was monitored throughout recording. Membrane currents were recorded in a whole-cell configuration using an Axopatch-1D amplifier and pCLAMP 10 software (Axon Instruments, Foster City, CA, USA), digitized, and stored on a computer disk for off-line analysis. Capsaicin and allylisothiocyanate (AITC)-induced current responses were recorded >5 min after the establishment of whole-cell configuration. Capsaicin current was induced by perfusion of 1 μM capsaicin for 15 s. More than 5 min after the capsaicin current disappeared, AITC currents were induced by perfusion of 500 μM AITC for 30 s. The extracellular solution was perfused at 3–4 mL/min.

### Behavioral Tests

Pain responses to noxious heat stimuli were assessed in 8-week-old C57BL/6J male mice (body weight; 20–24 g) by hot plate test. Mice were habituated to placement in experimental room for at least 30 min and on disconnected hot plate for 15 min before the test. The heated surface of the plate was kept at a temperature of 52 ± 0.2°C (Model-DS 37, Ugo Basile, Comerio, Italy), as measured by a built-in digital thermometer, and verified by a surface thermometer. Mice were placed on the hot plate and surrounded by a clear acrylic cage (200-mm diameter, 130 mm height). The latency to respond was measured as the time required for the mouse to lick, flick or bite its hind paw, or jump. After any of these responses, the mouse was immediately removed from the hot plate and returned to its home cage. Failure to respond within 20 s was grounds for removal from the test, a cutoff established to prevent tissue damage. All drugs were dissolved in saline and injected at 10 μl/paw. Control mice were injected with saline. Testing was performed three times in each mouse; 30 min prior to the intraplantar drug injection, 15 min after drug injection and 4 h after drug injection. To evaluate the changes in latency, the latency 30 min prior to the drug injection was subtracted from those observed 15 min, and 4 h after the treatment. Positive and negative values implied reduction and elevation of noxious heat sensitivity, respectively.

### Drugs

Glutamate, quisqualate, capsaicin, AITC and actinomycin D were obtained from Sigma-Aldrich (St. Louis, MO, USA); 7-(hydroxyimino)cyclopropa[b]chromen-1a-carboxylate ethyl ester (CPCCOEt), 2-methyl-6-(phenylethynyl)pyridine (MPEP), dihydroxyphenylglycine (DHPG), 2,3-dioxo-6-nitro-1,2,3,4-tetrahydrobenzo[f]quinoxaline-7-sulfonamide (NBQX) and 1-[6-[[(17β)-3-Methoxyestra-1,3,5(10)-trien-17-yl]amino]hexyl]-1H-pyrrole-2,5-dione (U73122) were obtained from Tocris Cookson (Bristol, UK); 2-(2-Amino-3-methoxyphenyl)-4H-1-benzopyran-4-one (PD98059) were obtained from Wako (Osaka, Japan); 3-[3-[2,5-Dihydro-4-(1-methyl-1H-indol-3-yl)-2,5-dioxo-1H-pyrrol-3-yl]-1H-indol-1-yl]propyl carbamimidothioate (Ro31–8220) and 4-[5-(4-Fluorophenyl)-2-[4-(methylsulfonyl)phenyl]-1*H*-imidazol-4-yl] pyridine (SB203580) were obtained from Enzo Life Sciences (East Farmingdale, NY, USA). For calcium imaging, capsaicin (0.5 μM), AITC (200 μM), glutamate (3, 10 and 30 μM), quisqualate (10 μM), NBQX (10 μM), CPCCOEt (100 μM), MPEP (50 μM), DHPG (50 μM), U73122 (2 μM), Ro 31–8220 (100 nm), PD98059 (50 μM), SB203580 (1 μM), and actinomycin D (5 μg/mL) were dissolved in ACSF. For whole-cell recording, capsaicin (1 μM), AITC (500 μM), and quisqualate (10 μM) were dissolved in ACSF. In the hot plate test, quisqualate (1 and 2 mM), DHPG (1 and 3 mM), CPCCOEt (1.25 mM), MPEP (2.5 mM), PD98059 (500 μM), and SB203580 (10 μM) were dissolved in 0.9% saline containing 10% DMSO.

### Statistical Analysis

Data were analyzed with SigmaPlot 13.0 Software (Systat Software Inc., San Jose, CA, USA). Results are expressed as mean ± standard error of the mean (SEM). The number of the cells examined are represented by “*n*”. Parametric and non-parametric data were assessed using the one-way analysis of variance (ANOVA) and Kruskal–Wallis test, respectively. Chi-squared tests were used to assess changes in the proportion of capsaicin-sensitive neurons. A *p*-value of < 0.05 was considered statistically significant.

## Results

### Long-Term Activation of mGluR1/5 Increases the Proportion of Capsaicin-Sensitive Neurons

**Figure 1:**
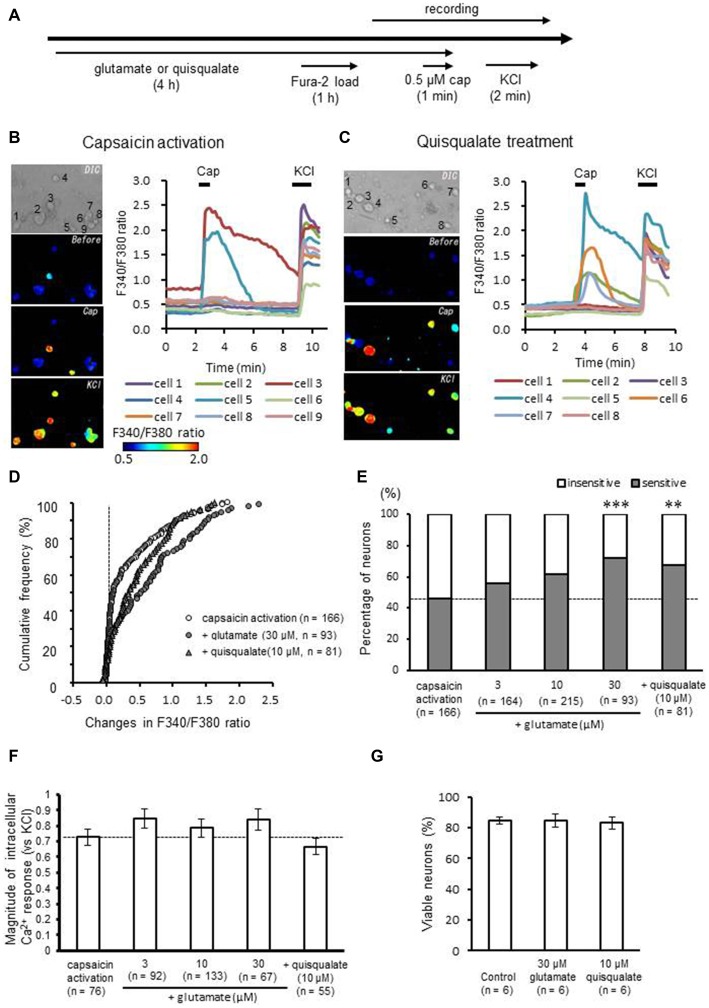
**Effects of long-term application of glutamate and quisqualate on capsaicin-induced intracellular calcium elevation. (A)** Experimental design for the recording of capsaicin-induced intracellular calcium elevation after long-term application of glutamate and quisqualate using Fura-2 AM dye. Representative images of F340/F380 ratio before and after capsaicin (cap; 0.5 μM) and KCl (50 mM) perfusion using Fura-2 AM in **(B)** control and **(C)** quisqualate-treated neurons. **(D)** The cumulative curve of calcium response induced by capsaicin in dorsal root ganglion (DRG) neurons in 30 μM glutamate- (closed circles) and 10 μM quisqualate-treated groups (closed triangles). The *X* axis represents changes observed in F340/F380 by capsaicin in each recorded neuron. The *Y* axis represents the cumulative frequency of neurons arranged in ascending order of capsaicin responses. A vertical dashed line represents *x* = 0. **(E)** The change in the proportion of capsaicin-sensitive (gray) and -insensitive (white) neurons. **(F)** Bar graph shows magnitude of capsaicin-induced intracellular Ca^2+^ responses normalized to KCl. Values are represented as mean ± SEM of whole capsaicin-sensitive neurons in each group. **(G)** Changes in the percentage of viable neurons after glutamate or quisqualate treatment. **p < 0.01, ***p < 0.001 against control.

To evaluate the effect of long-term activation of glutamate receptors on TRPV1 responses, cultured DRG neurons were incubated for 4 h in ACSF containing glutamate (30 μM) or quisqualate (10 μM), AMPA receptor and mGluR1/5 agonist (Figure 1A). TRPV1-mediated responses were induced by perfusion of 0.5 μM capsaicin, a TRPV1 agonist. Responses were assessed by measuring changes in intracellular calcium concentration with Fura-2 AM. Perfusion of capsaicin caused clear intracellular calcium elevation in a subset of DRG neurons (Figure 1B). Long-term exposure to quisqualate appeared to produce an increase in the proportion of DRG neurons sensitive to capsaicin (Figure 1C). Figure 1D shows the changes in the cumulative curve for intracellular calcium responses induced by capsaicin after glutamate or quisqualate treatment. In the figure, the *Y* axis represents the cumulative frequency of recording neurons arranged in ascending order to capsaicin responses (ΔF340/F380). In control group, more than half of the DRG neurons (54.2%) showed little to no response to capsaicin (ΔF340/F380 ratio < 0.15). However, glutamate and quisqualate treatment increased the proportion of capsaicin-sensitive neurons to 72.0 and 67.9%, respectively. Data are summarized in Figure 1E. The proportion of capsaicin-sensitive neurons significantly increased after treatment with glutamate (30 μM; 67 out of 93 neurons, 72.0%, *p* < 0.001 compared to control group) or quisqualate (10 μM; 55 out of 81 neurons, 67.9%, *p* < 0.01), while 76 out of 166 neurons (45.8%) responded to capsaicin in control DRG neurons. This increase occurred in a concentration-dependent manner after treatment with glutamate (3–30 μM, Figure 1E). Although we analyzed the magnitude of capsaicin-induced maximal response normalized to KCl, there was no significant difference in the amplitudes for either of ligand concentration (*p* = 0.069: Figure 1F). We performed tripan blue staining after 30 μM glutamate or 10 μM quisqualate treatment for 4 h (Figure 1G). Long-term treatment with these drugs did not cause neuronal death in the DRG culture.

**Figure 2:**
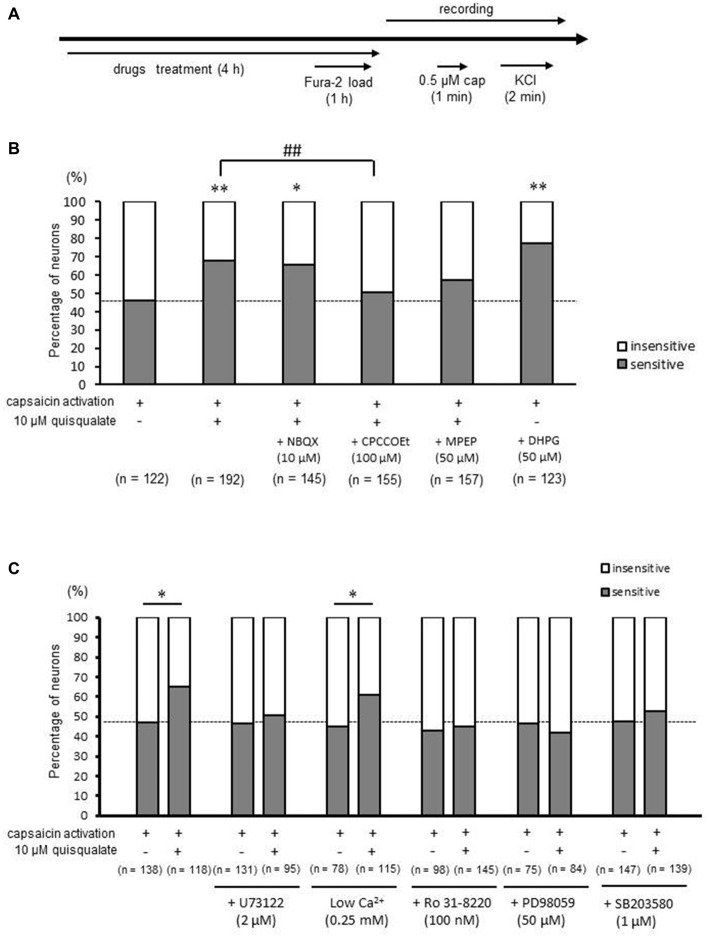
**Effects of selective glutamatergic receptors antagonists and intracellular signaling pathway inhibitors on quisqualate-induced increase in capsaicin-sensitive neurons. (A)** Experimental design for the recording of capsaicin-induced calcium elevation. **(B)** The change in the proportion of capsaicin-sensitive (gray) and -insensitive (white) neurons in presence of selective glutamate receptor inhibitors with quisqualate. **(C)** The change in the proportion of capsaicin sensitive (gray) and insensitive (white) neurons in the presence of intracellular signaling pathway inhibitors with quisqualate. *p < 0.05, **p < 0.01 against control. ^##^*p* < 0.01 against quisqualate-treated alone.

In the next experiment, DRG neurons were treated with 10 μM quisqualate for 4 h, followed by recording capsaicin responses in the absence of quisqualate (Figure 2A). An increase in proportion of capsaicin-sensitive neurons was observed even after the washout of quisqualate (Figure 2B). The increased proportion of capsaicin-sensitive neurons associated with quisqualate (from 47.5% in control to 67.7% following quisqualate application) was significantly antagonized by treatment with 100 μM of the selective mGluR1 antagonist CPCCOEt, (78 out of 155 neurons, 50.3%, *p* < 0.01), but not by 50 μM of the selective mGluR5 antagonist MPEP (90 out of 157 neurons, 57.32%, *p* = 0.177; Figure 2B). However, treatment with NBQX (10 μM), a selective AMPA receptor antagonist, did not affect the quisqualate-related increase in capsaicin-sensitive neurons (95 out of 145 neurons, 65.5%, *p* = 1.00). In addition, DHPG, a selective mGluR1/5 agonist, significantly increased the proportion of capsaicin-sensitive neurons (95 out of 123 neurons, 77.2%, *p* < 0.01; Figure 2B). These results indicate that the increase in proportion of capsaicin-sensitive cells was caused by mGluR1 activation, and only partially by activation of mGluR5.

### Increases in the Proportion of Capsaicin-Sensitive Neurons by mGluR1/5 Activation was Mediated by PLC, PKC, MEK and p38 MAPK

Metabotropic glutamate receptors 1/5 are involved in numerous signaling pathways. The increase in the proportion of capsaicin-sensitive neurons induced by quisqualate was suppressed by treatment with 2 μM U73122 (control; 61 out of 131 neurons, 46.6% vs. quisqualate; 48 out of 95 neurons, 50.5%, *p* = 0.650), a PLC inhibitor, or 100 nm Ro31–8220 (control; 42 out of 98 neurons, 42.9% vs. quisqualate; 65 out of 145 neurons, 44.8%, *p* = 0.864), a protein kinase C (PKC) inhibitor (Figure 2C). In addition, the quisqualate-induced increase in capsaicin-sensitive neurons was inhibited by 50 μM PD98059 (control; 35 out of 75 neurons, 46.7% vs. quisqualate; 35 out of 84 neurons, 41.7%, *p* = 0.636), a mitogen/extracellular signal-regulated kinase (MEK) inhibitor, or 1 μM SB203580 (control; 70 out of 147 neurons, 47.6% vs. quisqualate; 73 out of 139 neurons, 52.5%, *p* = 0.478), a p38 mitogen-activated protein kinase (MAPK) inhibitor. To elucidate the contribution of extracellular calcium influx, quisqualate was applied under zero calcium conditions. However, it was impossible to maintain cultured DRG neurons for 4 h. Therefore, DRG neurons were treated with quisqualate in a low calcium solution (0.25 mM) for 4 h, followed by capsaicin at normal concentration of calcium (2.5 mM). Consequently, a quisqualate-induced increase in capsaicin-sensitive neurons was observed (control; 35 out of 78 neurons, 44.9% vs. quisqualate; 70 out of 115 neurons, 60.9%, *p* < 0.05). Therefore, it is suggested that long-term application of mGluR1/5 activates PLC, PKC, MEK and p38 MAPK irrelevant to extracellular calcium influx, producing an increase in capsaicin-sensitive neurons.

### Activation of mGluR1/5 Increases the Proportion of Capsaicin-Sensitive Neurons in a Time-Dependent Manner

**Figure 3:**
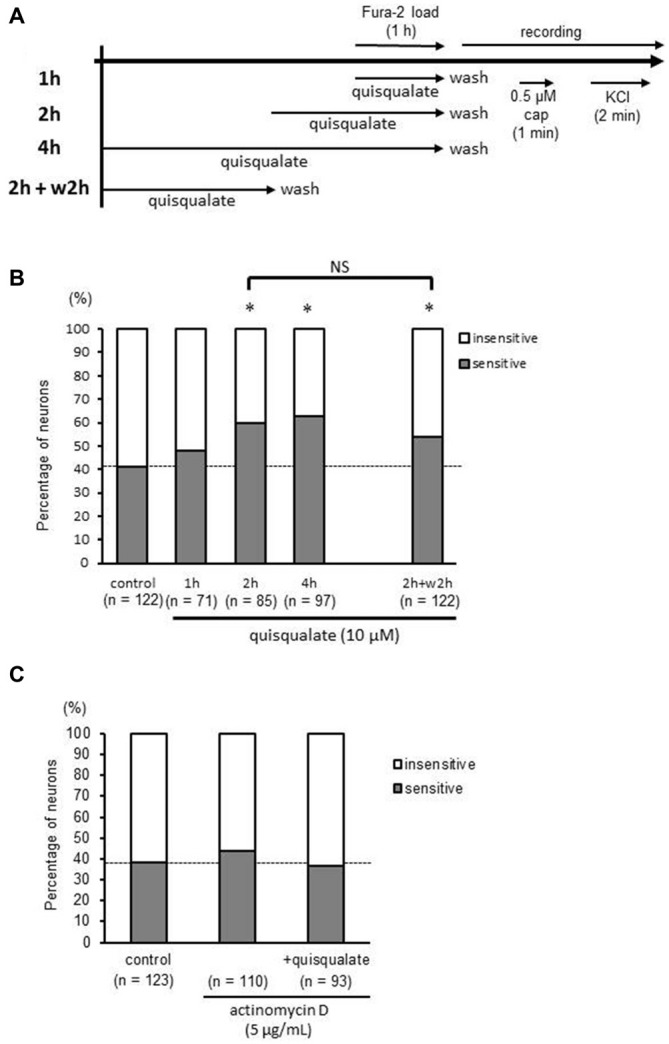
**Time-dependent increase in capsaicin-sensitive neurons induced by quisqualate is blocked by a transcription inhibitor. (A)** Experimental design for the recording of capsaicin (cap; 0.5 μM)-induced calcium elevation to determine time-dependency of quisqualate effect. **(B)** Effects of quisqualate treatment duration on the proportion of capsaicin-sensitive (gray) and -insensitive (white) neurons. **(C)** Change in the proportion of capsaicin-sensitive (gray) and -insensitive (white) neurons after treatment of quisqualate (4 h) in presence of actinomycin D. *p < 0.05 against control. NS, non-significant.

To examine the time-dependency of the observed increases in the proportion of capsaicin-sensitive neurons, 10 μM quisqualate was applied to the DRG culture for 1–4 h (Figure 3A). Quisqualate time-dependently increased number of capsaicin-sensitive neurons, and the increases became significant for drug application durations lasting over 2 h (51 out of 85 neurons, 60.0%, *p* < 0.05; Figure 3B). This increase in the proportion of capsaicin-sensitive neurons was sustained even after 2 h of washes and incubation in the absence of quisqualate (67 out of 122 neurons, 54.9%, *p* < 0.05; Figure 3B). We next examined whether the observed increase in capsaicin-sensitive neurons was related to gene expression. Actinomycin D (5 μg/mL), a transcription inhibitor, completely inhibited the quisqualate-induced increase in capsaicin-sensitive neurons (actinomycin D alone: 48 out of 110 neurons, 43.6%, vs. actinomycin D + quisqualate: 34 out of 93 neurons, 36.6%; *p* = 0.379; Figure 3C).

### Increases in the Proportion of Capsaicin-Sensitive Neurons in Transient Receptor Potential Ankyrin Type 1 (TRPA1)-Expressing Neurons

**Figure 4:**
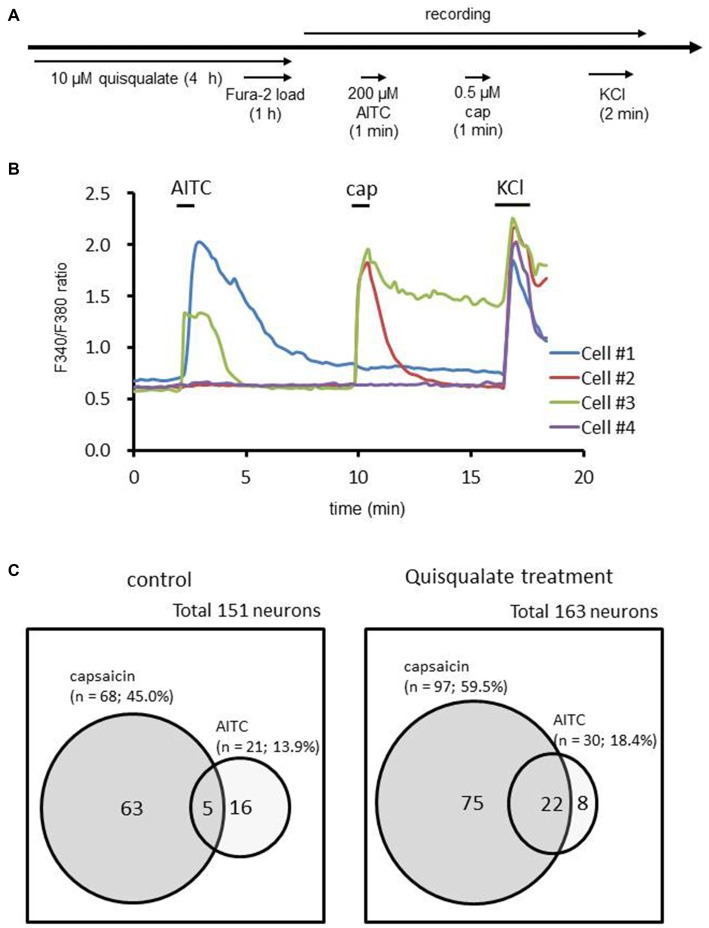
**Changes in the proportion of allylisothiocyanate (AITC)-sensitive neurons that respond to capsaicin. (A)** Experimental design for recording of AITC (200 μM)- and capsaicin (cap; 0.5 μM)-induced calcium elevation after long-term quisqualate application using Fura-2 AM. **(B)** Time course of intracellular calcium responses in control culture. Cell #1; Response to AITC. Cell #2; Response to capsaicin. Cell #3; Response to both AITC and capsaicin. Cell #4; No response to either AITC or capsaicin. **(C)** The change in the cell population after long-term application of quisqualate.

TRPA1 as well as TRPV1 are recognized as multiple irritant sensors in the sensory neuron that contribute to the pain response (Guimaraes and Jordt, 2007; Zurborg et al., 2007). Recent studies revealed that TRPA1 interacts with TRPV1 and contributed to the development of inflammatory pain (Akopian, 2011). To clarify the relationship with TRPA1-positive neurons in the increase of the functional TRPV1-expressing neurons population, we examined the proportion of capsaicin-sensitive neurons in TRPA1-expressing neurons before and after chronic application of quisqualate. To identify DRG neurons expressing TRPA1, AITC (a TRPA1 agonist) was perfused before capsaicin application (Figure 4A). Figure 4B shows the representative calcium mobilization detected with Fura-2 loaded in DRG neurons. Application of AITC (200 μM) and/or capsaicin (0.5 μM) induced intracellular calcium elevation in a distinct population of DRG neurons. The proportions of neurons that responded to AITC and/or capsaicin are shown in Figure 4C. Treatment with quisqualate for 4 h increased the proportion of capsaicin-sensitive neurons from 45.0% (control; 68 out of 151 neurons) to 59.5% (quisqualate; 97 out of 163 neurons, *p* < 0.05), in contrast, there are no significant changes in the proportion of AITC-sensitive neurons (control; 13.9% vs. quisqualate; 18.4%, *p* = 0.34). Among the neurons that responded to AITC, the increase in the capsaicin-sensitive population was drastic (23.8%; 5 out of 21 neurons to 73.3%; 22 out of 30 neurons, *p* < 0.01). These results implicate that the increase in capsaicin-sensitive population was mainly derived from AITC-sensitive neurons.

### Long-Term Application of Quisqualate Increases Capsaicin-Induced Current Density in AITC-Sensitive Neurons

**Figure 5:**
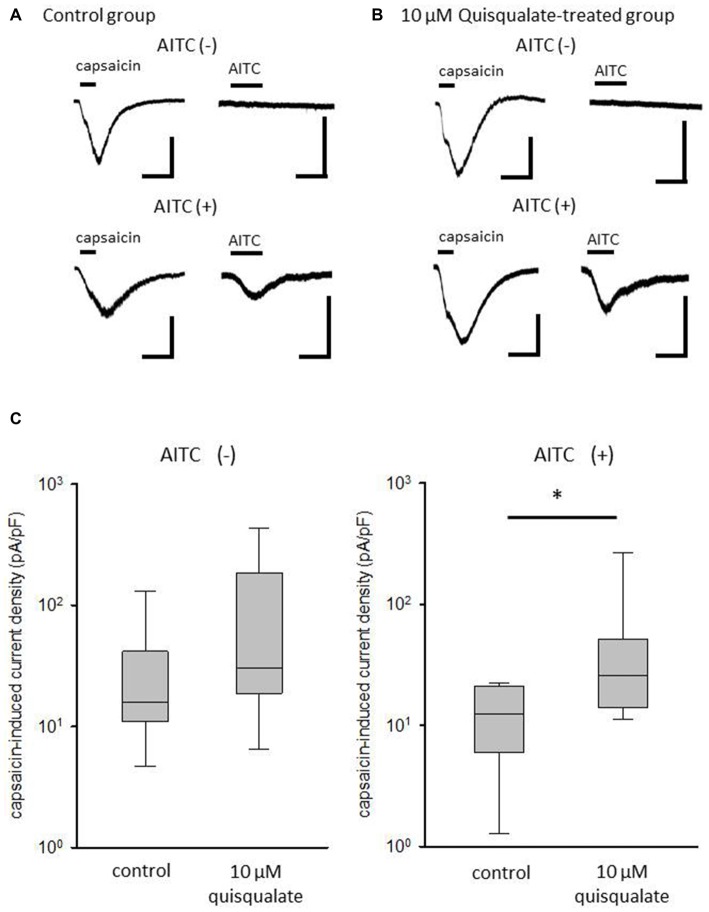
**Changes in capsaicin-induced current after long-term treatment with quisqualate.** Representative traces of TRPV1 current induced by 1 μM capsaicin and transient receptor potential ankyrin type 1 (TRPA1) current induced by 500 μM AITC in **(A)** control and **(B)** quisqualate-treated DRG neurons. The membrane potential was held at -70 mV. *Top*, representative current traces from a neuron responsive to capsaicin, but not AITC. *Bottom*, traces from a neuron responsive to both capsaicin and AITC. Scale bars = 500 pA, 30 s. **(C)** The difference in current density of capsaicin response between control and quisqualate-treated neurons in AITC-insensitive (left) and AITC-sensitive neurons (right). Box and whisker plots show maximum, minimum, upper and lower quartiles, and median values. *p < 0.05 against control.

To identify changes in the current density of TRPV1 after long-term activation of mGluR1/5, capsaicin -induced current were measured in AITC-sensitive and -insensitive neurons after quisqualate treatment for 4 h (Figure 5). Whole-cell recordings were conducted on cultured DRG neurons, and the membrane potential was held at −70 mV. Capsaicin at a concentration of 1 μM was rapidly perfused into the recording chamber for 15 s and then washed out with ACSF for more than 2 min. To check AITC responsiveness, 500 μM AITC was perfused into the chamber for 30 s. Representative recordings from control and quisqualate-treated DRG neurons are shown in Figures 5A,B, respectively. Although the current response to capsaicin in AITC-sensitive DRG neurons tends to be smaller than in AITC-insensitive DRG neurons (Figure 5A), quisqualate seems to increase the current response of capsaicin in AITC-sensitive neurons (Figure 5B). Quisqualate treatment caused significant increase in current density induced by capsaicin in AITC-sensitive neurons (*p* < 0.05; Figure 5C), but not AITC-insensitive neurons (*p* = 0.081; Figure 5C).

### Intraplantar Injection of Quisqualate Causes Hyperalgesia via mGluR Activation

**Figure 6:**
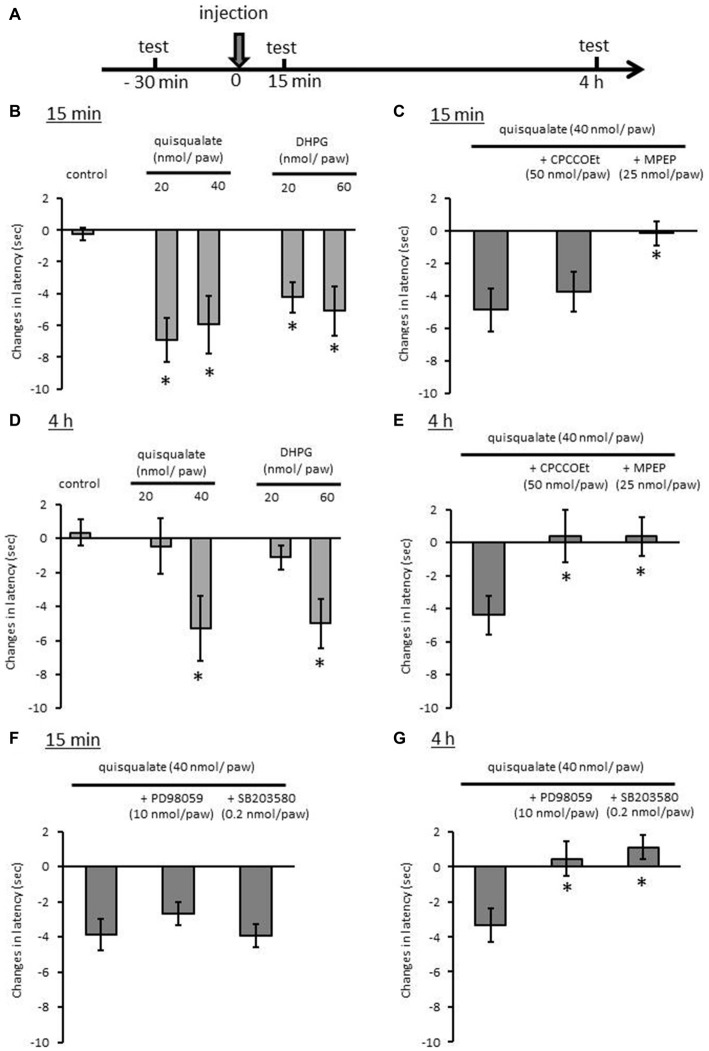
**Effects of intraplantar injection of glutamatergic drugs in the hot plate test in mice. (A)** Experimental design for evaluation of heat sensitivity 15 min and 4 h after drug injection. Changes in latency to shake, lick, or jump after placement on hot plate (52.0 ± 0.2°C) 15 min **(B)** or 4 h **(D)** after injection of quisqualate and DHPG. Effects of selective mGluR1 and mGluR5 antagonists on quisqualate-induced hyperalgesia 15 min **(C)** or 4 h **(E)** after the injection. Effects of selective MEK and p38 MAPK inhibitors on quisqualate-induced hyperalgesia 15 min **(F)** or 4 h **(G)** after the injection. Eight mice were used in each group. Values represent mean ± SEM. *p < 0.05 against control.

We reported that TRPV1 activation is necessary to pain response to noxious heat in hot plate test, because 5′-iodoresiniferatoxin, a selective TRPV1 antagonist inhibits the heat response (Masuoka et al., 2015b). In a previous report, we showed that quisqualate at a dose of 4 nmol/paw significantly decreased the latency of heat response 15 min after the treatment. To test the long-term activation of mGluR1/5 on noxious heat sensitivity, higher doses of quisqualate or DHPG were injected into the foot soles of mice. Hot plate tests were subsequently performed 15 min and 4 h after the injection (Figure 6A). Fifteen minutes after quisqualate administration, we observed a significant decrease in the latency to shake, lick, or jump on the hot plate at doses of 20 and 40 nmol/paw (*p* < 0.05; Figure 6B). DHPG also decreased the latency at doses of 20 and 40 nmol/paw (*p* < 0.05). The quisqualate-induced heat hyperalgesia was abolished by MPEP (25 nmol/paw;* p* < 0.05) but not by CPCCOEt (50 nmol/paw; *p* = 0.986; Figure 6C). Decreased response latency persisted even 4 h after the quisqualate and DHPG injection at doses of 40 and 60 nmol/paw, respectively (*p* < 0.05; Figure 6D). This effect at 4 h was completely antagonized by CPCCOEt (*p* < 0.05) or MPEP (*p* < 0.05; Figure 6E). Finally, the effects of PD98059 and SB203580 on quisqualate (40 nmol/paw) induced heat hyperalgesia were examined. Decreased response latency 4 h after the quisqualate injection was antagonized by the concurrent injection of PD98059 (10 nmol/paw;* p* < 0.05) or SB203580 (0.2 nmol/paw;* p* < 0.05, Figure 6G). There was no significant effect on decreased latency 15 min after the injection by either kinase inhibitor (Figure 6F).

## Discussion

In the present study, we evaluated TRPV1 function by measuring intracellular calcium responses to capsaicin in cultured DRG neurons. Application of non-selective glutamatergic receptor agonists for 4 h increased the proportion of capsaicin-sensitive neurons. This effect was inhibited by the administration of a selective mGluR1 antagonist and only partially by an mGluR5 antagonist. These results indicate that long-term activation of mGluR1 and mGluR5 increase the population of DRG neurons expressing functional TRPV1. In addition, this phenomenon is likely to depend on the expression of TRPV1 and/or proteins modulating TRPV1 sensitivity such as TRPV1b (Vos et al., 2006), and A-kinase anchoring protein (Jeske et al., 2009), because the enhanced neuron recruitment persisted up to 2 h following cessation of mGluRs activation and was completely inhibited in the presence of a transcription inhibitor, actinomycin D. It has been reported that mGluR1/5 activates several intracellular signaling molecules, phosphatidyl inositol 3 (PI3)-kinase, PKC, p38 MAPK, striatal-enriched protein tyrosine phosphatase (STEP), and caspase-3 (O’Riordan et al., 2006; Young et al., 2008; Chen et al., 2013). Kwon et al. (2014) reported that TRPV1 expression in DRG neurons is under the control of p38 MAPK phosphorylation in inflammatory sites. In present study, we also confirmed that the increase in capsaicin-sensitive neuron was suppressed by application of PLC, PKC, MEK and p38 MAPK inhibitors. Therefore, it was suggested that persistent activation of mGluR1/5 enhanced MEK and p38 MAPK pathways via PLC and PKC activation, resulted in increased numbers of DRG neurons expressing functional TRPV1. Recently, an inflammatory mediator prostaglandin E2, whose metabotropic receptors EP1–4 were expressed in DRG neurons, was found to facilitate the amount of TRPV1 expression, and the percentage of TRPV1-positive neurons in the rat neuropathic pain model (Ma et al., 2010). In addition, activation of the muscarinic acetylcholine receptor M2 decreased the TRPV1 transcript expression, and the number of capsaicin-responsive neurons in mice DRG (De Angelis et al., 2014). Therefore, metabotropic receptors might be one of the important regulators of functional TRPV1 expression in primary sensory neurons.

Although short-term stimulation of mGluR5 in cultured DRG neurons potentiated intracellular calcium influx via TRPV1 activation (Masuoka et al., 2015b), the magnitude of individual intracellular calcium elevation induced by capsaicin was not changed after long-term activation of glutamate receptors. These results suggest that the augmentation of the TRPV1-mediated intracellular response by mGluR5 disappeared after long-term stimulation of glutamate receptors. Direct phosphorylation of TRPV1 via PKC activation has been shown to acutely sensitize the TRPV1 response (Bhave et al., 2003), which is related to potentiation of TRPV1-mediated intracellular calcium responses by short-term stimulation of mGluR5. On the other hand, work in Xenopus oocytes has shown that the PKC activated by mGluR5 can simultaneously phosphorylate several targets of mGluR5a (S613, S881, S890 etc.), resulting in mGluR5 desensitization (Gereau and Heinemann, 1998). Therefore, the persistent stimulation of mGluR5 may desensitize acute mGluR5-TRPV1 interaction.

It has been shown that TRPV1 expression in nociceptive DRG neurons is increase in peripheral neuropathy in rodents (Hudson et al., 2001; Rashid et al., 2003; Biggs et al., 2007; Kim et al., 2008). For instance, TRPV1 protein in glabrous skin is overexpressed in Fabry disease model mice showing peripheral neuropathy (Lakomá et al., 2014). Kwon et al. (2014) found that heat hyperalgesia in inflammatory pain correlates to expression of TRPV1, a molecule working as a sensor of noxious heat in sensory neurons. Inflammatory pain induced by complete Freund’s adjuvant increases artemin, resulting in increase of TRPV1/A1-positive DRG neurons (Ikeda-Miyagawa et al., 2015). In the present study, we show that the population of functional TRPV1-expressing neurons dramatically increased in TRPA1-expressing DRG neurons, and TRPV1 current density significantly increased in TRPA1-expressing neurons. The TRPA1 channel was originally proposed to be a multiple irritant sensor and a noxious cold sensor (Guimaraes and Jordt, 2007; Zurborg et al., 2007). Thus, it is compelling that the increase in TRPV1 activity in TRPA1-positive neurons augments centripetal flow of inputs associated with pain, even when rodents are exposed to the same noxious heat stimuli.

TRPV1-TRPA1 interactions in peripheral neurons are important for adaptive development of inflammatory pain system (Akopian, 2011). Bandell et al. (2004) reported that one of the dominant inflammatory mediators, bradykinin, could indirectly activate TRPA1, likely via production of diacylglycerol. It has also been known that the pharmacological desensitization of TRPA1-mediated responses in sensory neurons lacking TRPV1 is more pronounced than in neuron expressing TRPV1 (Akopian et al., 2007). Taken together, these lines of evidence suggest that the co-expression of TRPV1 and TRPA1 as a consequence of mGluR1/5 activation may lead to a slower desensitization of bradykinin responses in sensory neurons, and this, in turn, may enhance the development of inflammatory hyperalgesia.

Disruption of protein kinase A (PKA) or PKC-mediated TRPV1 sensitization attenuates λ-carrageenan-induced heat hyperalgesia (Fischer et al., 2013). In addition, increase of TRPV1/TRPA1 expression induced artemin, a neurotrophic factor, caused heat hyperalgesia (Ikeda-Miyagawa et al., 2015). We employed a hot plate test to test whether the increase in functional TRPV1-expressing neurons induced by mGluR1/5 affects noxious heat sensitivities in the periphery. Intraplantar injections of high-dose quisqualate induced persistent heat hyperalgesia for 4 h in the present study. Hyperalgesia 15 min after the injection was attenuated by mGluR5 antagonist, but not by mGluR1 antagonist. This result confirms that the acute phase of hyperalgesia is caused by augmentation of the TRPV1 response by mGluR5, without changes in the proportion of TRPV1-expressing neurons, as we previously reported (Masuoka et al., 2015b). On the other hand, hyperalgesia 4 h after the injection was inhibited by both mGluR1 and mGluR5 antagonists. In addition, the MEK and p38 MAPK inhibitors selectively attenuated hyperalgesia 4 h after the injection. This suggests that chronic heat hyperalgesia is related to the increase in the proportion of functional TRPV1-expressing neurons following mGluR1 and mGluR5 activation.

In conclusion, long-term activation of mGluR1/5 produces an increase in the number of DRG neurons expressing functional TRPV1, which may contribute to the development of chronic heat hyperalgesia. Persistent elevation of glutamate release in the periphery may be crucial for understanding sensory disorders induced by inflammation and tissue damage.

## Author Contributions

TM designed and performed the research, analyzed the data and wrote the article. MK, JY and NI performed research and analyzed data. JY, TI, IM, NK and MN wrote and revised the article.

## Conflict of Interest Statement

The authors declare that the research was conducted in the absence of any commercial or financial relationships that could be construed as a potential conflict of interest. The reviewer LC and handling Editor declared their shared affiliation, and the handling Editor states that the process nevertheless met the standards of a fair and objective review. The reviewer FF and handling Editor declared their shared affiliation, and the handling Editor states that the process nevertheless met the standards of a fair and objective review.
